# 2048. Molecular Characterization and Resistance Factors of Circulating *Acinetobacter baumannii* isolates in South-East Michigan

**DOI:** 10.1093/ofid/ofac492.1670

**Published:** 2022-12-15

**Authors:** Hosoon Choi, Jing Xu, Munok Hwang, Chetan Jinadatha, Thanuri Navarathna, Landon Ashby, Morgan Bennett, Keith S Kaye, Sorabh Dhar, Piyali Chatterjee

**Affiliations:** Central Texas Veterans Health Care System, Temple, Texas; Central Texas Veteran Health Care System, Temple, Texas; Central Texas Veterans Health Care System, Temple, Texas; Central Texas Veterans Health Care System, Temple, Texas; Central Texas Veterans Health Care System, Temple, Texas; Central Texas Veterans Health Care System, Temple, Texas; Central Texas Veterans Health Care System, Temple, Texas; Rutgers - Robert Wood Johnson Medical School, New Brunswick, New Jersey; Wayne State University/Wayne Health, John Dingell VAMC, Detroit, Michigan; Central Texas Veterans Health Care System, Temple, Texas

## Abstract

**Background:**

Carbapenem-resistant *Acinetobacter baumannii* (*CRAb*) is increasing due to widespread use of antibiotics. Multidrug resistant (MDR) *CRAb* is a major threat to public health as treatment options are limited. The objective of this study is to elucidate the molecular epidemiology of circulating antibiotic resistance genes causing MDR *CRAb* infections by using a combination of whole-genome Multi-Locus Sequence Typing (wgMLST) and antibiotic susceptibility phenotyping.

**Table 1.**
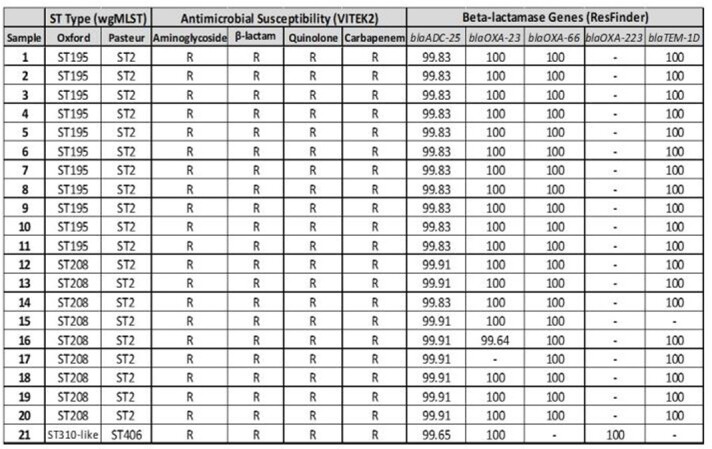
Molecular characterization of MDR CRAb isolates

**Methods:**

Bacterial isolates were derived from cultures taken from subjects 48 hours following admission as part of routine clinical care for patients between 2017-2020. Isolates were obtained from 16 hospital units (both ICU and non-ICU) across two hospitals in the Detroit area. Whole Genome Sequencing (WGS) was performed using Illumina MiniSeq or Nextseq. WgMLST analysis was performed using BioNumerics software v7.6. ResFinder software was used for analysis of antibiotic resistance genes. Isolates underwent antibiotic susceptibility testing using a broth microdilution method (VITEK2) and Clinical & Laboratory Standards Institute (CLSI) minimum inhibitory concentration (MIC) cut offs.

**Results:**

Out of the 95 total isolates, 51(54%) were *CRAb* isolates and of the *CRAb* isolates, 21(41%) were MDR *CRAb*. WgMLST identified that majority of the circulating MDR *CRAb* isolates belonged to ST2^Pas^ (ST195^Ox^ and ST208^Ox^) based on CDC definitions (Table 1). MDR *CRAb* isolates were resistant to 3 different classes of antibiotics including aminoglycosides, fluroquinolones and β-lactams. β-lactamase genes present include (blaADC-25, blaOXA-23, blaOXA-66 and blaTEM1D) for both ST195^Ox^ and ST208^Ox^ and (blaADC-25, blaOXA-23 and blaOXA-223) for ST406^Pas^ (ST310^Ox^). Among the patients with MDR *CRAb* infections, most were males with respiratory infections in a non-ICU setting.

**Conclusion:**

The study demonstrated a high proportion of isolates belonged to ST2 ^Pas^ carrying multiple beta-lactamase genes including blaOXA-23 gene. ST406^Pas^ might be an emerging lineage carrying the blaOXA-23 gene. In addition to stringent infection control measures, continuous surveillance is recommended in limiting the spread of MDR *CRAb* isolates in the healthcare settings.

**Disclosures:**

**Chetan Jinadatha, MD, MPH**, AHRQ R01 Grant-5R01HS025598: Grant/Research Support|EOS Surfaces: Copper Coupons and materials for testing **Keith S. Kaye, MD, MPH**, Allecra: Advisor/Consultant|GlaxoSmithKline plc.: Receiving symposia honoraria|GlaxoSmithKline plc.: GlaxoSmithKline plc.-sponsored study 212502|Merck: Advisor/Consultant|qpex: Advisor/Consultant|Shionogi: Grant/Research Support|Spero: Advisor/Consultant **Piyali Chatterjee, PhD**, AHRQ Grant # 1R03HS027667-01: Grant/Research Support|AHRQ Grant # 1R03HS027667-01: Central Texas Veterans Health Care System.

**Table 1.** Molecular characterization of MDR CRAb isolates

*The numbers indicate % of sequence identity match for each Beta-lactamase gene.

